# Transactions of the American Medical Association

**Published:** 1854-01

**Authors:** 


### TRANSACTIONS OF THE AMERICAN MEDICAL ASSOCIATION.—Vol. vi.—1853.

From a cursory examination of the volume of Transactions of the American Medical Association, for 1853, it appears to us to compare favorably with the volumes that have preceded it. Having been but recently received, we have not, as yet, been able to give to the perusal of the several papers which it contains, that time and attention which they claim. We are not, therefore, prepared to offer, at present, opinions formed after a critical examination of their merits respectively. This we may do hereafter. But, in the mean time, we should be glad to exert any influence within our power to extend the circulation of the work. Every

American practitioner of medicine, as it seems to us, should feel bound to procure it merely as an act of encouragement to the Association in her efforts in behalf of a national medical literature. But the several volumes have intrinsic recommendations, which should secure for them a place in the library of the intelligent physician. They comprise a mass of information pertaining to the various departments of medical science, which renders them highly valuable for reference, and a convenient means of keeping up with the progress of scientific investigations. It is not very creditable to the medical profession of this country that the American Association, in the distribution of the previous publications, was met with only a moderate patronage; and we learn with regret and mortification that in the State of Kentucky the encouragement, thus far, has been quite insignificant. We are credibly informed that, while in the small State of Connecticut there are some sixty subscribers to the Transactions, in the State of Kentucky there are not over a dozen! We trust our Kentucky readers will agree with us that the honor of the State is in some measure involved in this matter, and will find in this consideration an additional inducement to add their names to the list of the patrons of the annual publications.

The present volume, in size, with one exception, is equal to any of its predecessors. It comprises 869 pages. In typography, the appearance of all the volumes is uniform; but an important improvement was adopted at the last meeting, viz—furnishing them bound in cloth, in the place of mere paper covers.

We subjoin the table of contents, by which our readers can form some idea of the value of the work:—Minutes of the Sixth annual meeting of the Association, and the annual reports of the Treasurer and Committee of Publication. Address of Beverly R. Wellford, President of the Association. Report of the Committee on Medical Education, Z. Pitcher, M. D., chairman. Report of the Committee on Medical Literature, N. S. Davis, M. D., chairman. On the Agency of the Refrigeration produced by upward Radiation of Heat, as an exciting cause of Disease, Gouverneur Emerson, M. D., chairman. On the Results of Surgical Operations in Malignant Diseases, S. D. Gross, M. D., chairman. On the Epidemics of Tennessee and Kentucky, W. L. Sutton, M. D., chairman. On Acute and Chronic Diseases of the Neck of the Uterus, CHAS. D. MEIGS, M. D. On the Nature of Typhoidal Fevers, HENRY F. CAMPBELL, M. D., chairman. On Coxalgia, or Hip Disease, ALDEN MARCH, M. D. On the Surgical Treatment of Morbid Growths within the Larynx, GURDON BUCK, M. D. On the Sympathetic Nerve in Reflex Phenomena, HENRY F. CAMPBELL, M. D. Prize essay—On the Surgical Treatment of certain Fibrous Tumors of the Uterus, heretofore considered beyond the resources of art, WASHINGTON L. ATLEE, M. D., of Philadelphia, Pa. Prize essay—On the Cell, its Physiology, Pathology, and Philosophy, as deduced from original observations, to which is added its History and Criticism, WALDO I. BURNETT, M. D., of Boston, Mass.

This volume, and the preceding volumes,* may be ordered by addressing the Treasurer of the Association, D. F. CONDIE, M. D., or through Lea & Blanchard, Booksellers, &c., of Philadelphia.

* Excepting Vol. IV, nearly all the copies of which were destroyed by fire.

